# Investigation of associations between *Piezo1* mechanoreceptor gain-of-function variants and glaucoma-related phenotypes in humans and mice

**DOI:** 10.1038/s41598-020-76026-0

**Published:** 2020-11-04

**Authors:** Sally L. Baxter, William T. Keenan, Argus J. Athanas, James A. Proudfoot, Linda M. Zangwill, Radha Ayyagari, Jeffrey M. Liebmann, Christopher A. Girkin, Ardem Patapoutian, Robert N. Weinreb

**Affiliations:** 1 Hamilton Glaucoma Center, Viterbi Family Department of Ophthalmology, and Shiley Eye Institute, University of California San Diego (UCSD), 9415 Campus Point Drive, MC0946, La Jolla, CA 92093 USA; 2 Health Department of Biomedical Informatics, UCSD, La Jolla, CA USA; 3 Scripps Research Institute, La Jolla, CA USA; 4 Bernard and Shirlee Brown Glaucoma Research Laboratory, Edward S. Harkness Eye Institute, Columbia University Irving Medical Center, New York, NY USA; 5 Department of Ophthalmology and Vision Sciences, Callahan Eye Hospital, University of Alabama at Birmingham, Birmingham, AL USA

**Keywords:** Genetics, Eye diseases, Translational research

## Abstract

Glaucoma disproportionately affects individuals of African descent. Prior studies of the PIEZO1 mechanoreceptor have suggested a possible role in glaucoma pathophysiology. Here, we investigated associations between a *Piezo1* gain-of-function variant common in individuals of African descent with glaucoma-related phenotypes. We analyzed whole genome sequences to identify *Piezo1* variants and their frequencies among 1565 human participants. For the most common variant (e756del), we compared phenotypes between heterozygotes, homozygotes, and wildtypes. Longitudinal mixed effects models of visual field mean deviation (MD) and retinal nerve fiber layer (RNFL) thickness were used to evaluate progression. Based on trends in the models, further investigation was conducted using *Piezo1* gain-of-function mice. About 30% of African descent individuals had at least one e756del allele. There were trends suggesting e756del was associated with higher IOPs, thinner RNFLs, lower optic nerve head capillary densities, and greater decreases in MD and RNFL thickness over time, but these did not reach statistical significance. Among mice, increased *Piezo1* activity was not significantly associated with IOP or retinal ganglion cell density. Our study confirms that the *Piezo1* e756del gain-of-function variant is a frequent polymorphism present in African descent individuals but is unrelated to examined differences in glaucoma phenotypes. Ongoing work is needed to elucidate the role of *Piezo1*-mediated mechanotransduction in glaucoma.

## Introduction

Glaucoma is the leading cause of irreversible blindness worldwide^1,2^ and is characterized by progressive degeneration of the retinal ganglion cells (RGCs) and visual field loss^3,4^. Current management is focused on reducing intraocular pressure (IOP), the only proven modifiable risk factor^3^.

Increasing knowledge of mechanoreceptors over the last decade may help shed light on pressure-sensitive biological processes. PIEZO1 and PIEZO2 are mechanosensitive ion channel proteins, whose encoding genes (*Piezo1* and *Piezo2*) were identified in 2010^5^. PIEZOs have been shown to have critical roles in many functions dependent on force sensation, such as touch^6–8^, proprioception^8,9^, baroreception^10^, red blood cell function^11,12^, and vascular development^13^. Several studies have suggested that *Piezo1* may potentially have a role in glaucoma pathophysiology. Choi et al.^14^ found that *Piezo1* is expressed in the mouse optic nerve head and may contribute to astrocytes’ responsiveness to traumatic or glaucomatous injury. Koser et al.^15^ demonstrated that RGC axon growth in *Xenopus* was aberrant when *Piezo1* was knocked down. Similarly, *Piezo1* was found to inhibit axon regeneration in *Drosophila*^16^. In human donor eyes, *Piezo1* was identified in the trabecular meshwork^17^.

Furthermore, recent analyses have demonstrated a remarkably high frequency of *Piezo1* gain-of-function mutations in people of African descent^11^, a population disproportionately affected by glaucoma^18^. The high frequency in this population is thought to result from a red blood cell dehydration phenotype associated with these mutations that confers protection against malarial infection^11^. Understanding other clinical phenotypes associated with these *Piezo1* mutations may help shed light on differential disease risk in African descent individuals. For glaucoma, this is particularly relevant, because African descent individuals experience glaucoma onset at an earlier age and develop blindness at higher rates^18–20^.

The aim of this study was to investigate associations between *Piezo1* variants and phenotypes relevant to glaucoma. We did this by analyzing genomic and clinical data from human participants in a large clinical research cohort and by conducting experimental studies in mouse models.

## Methods

### Study design

First, we investigated potential phenotypic associations with *Piezo1* genotype variants in 1565 participants from the African Descent and Glaucoma Evaluation Study (ADAGES) and the Diagnostic Innovations in Glaucoma Study (DIGS). The characteristics and examination protocols of ADAGES and DIGS have been described previously^21–24^. In brief, these cohorts included glaucoma patients and control participants without glaucoma recruited from five study centers in the United States. Participants provided written informed consent at enrollment after explanation of the nature and possible consequences of the study. The study was approved by the UCSD Institutional Review Board and adhered to the tenets of the Declaration of Helsinki. Second, we conducted experimental studies in mice to evaluate for associations between *Piezo1* genotypic variants with intraocular pressure and RGC density. All animal procedures were approved by the Institutional Animal Care and Use Committees of The Scripps Research Institute (TSRI) and adhered to the ARVO Statement for the Use of Animals in Ophthalmic and Vision Research.

### Whole genome sequencing

We analyzed whole genome sequences from the human participants to identify *Piezo1* genotypic variants (a1998v and e756del) and determine the frequencies of these alleles in this study population. These were chosen given their identification as gain-of-function variants concentrated within populations of African descent^11^. Whole genome sequencing and variant calling was conducted by Human Longevity, Inc, leveraging the ‘Issac’ pipeline (Illumina, Inc., San Diego, CA) using the hg38 reference genome^25^. Variants were extracted for the *Piezo1* gene region (chr16:88,715,338–88,785,220) using bcftools, biomaRt, and tabix, and then filtered to only include high confidence calls. Any single nucleotide polymorphisms (SNPs) that cause the a1998v mutation or the e756 deletion were tabulated for wildtype, heterozygous, and homozygous, then mapped to the respective patient identifiers.

Admixture genotype mapping was conducted to ensure accuracy over self-reported ancestry. We used a panel of 100,000 ancestry informative markers to determine the percentage of admixture for each individual. Each individual was then recharacterized based on the highest percentage of admixture ancestry. Subsequent analyses were limited to African descent individuals based on admixture mapping to remove race as a potential confounder of phenotypes.

### Human phenotypic associations

Because the a1998v variant was present at low frequency in the study population (7 of 1565, 0.45%), we focused subsequent analyses on the e756del variant in African descent individuals. We examined whether there were any significant differences in systemic phenotypes such as age, gender, blood pressure, and body mass index (BMI). We then evaluated whether the e756del variant was associated with a range of ocular phenotypes relevant to glaucoma based on data from the most recent documented visit. These included intraocular pressure (IOP), axial length, spherical equivalent of refractive error, and central corneal thickness (CCT). Data from Spectralis optical coherence tomography (OCT) imaging (Heidelberg Engineering, GmBH, Heidelberg, Germany) included retinal nerve fiber layer (RNFL) thickness from the optic nerve head circle scan and ganglion cell complex (GCC) thickness. Additional phenotypes included mean deviation (MD) on Humphrey Visual Field 24-2 perimetry testing (Carl Zeiss Meditech, Inc., Dublin, CA, USA) and capillary density as measured by optical coherence tomography angiography (OCTA) in both the macula and optic nerve (Optovue, Inc., Fremont, CA, USA). Genotype–phenotype associations were evaluated for all African descent individuals in aggregate and then additional analyses were performed stratified by glaucoma status (i.e. glaucoma only, healthy/non-glaucoma only).

To examine whether e756del may influence the rate of glaucoma progression, longitudinal analyses were performed examining changes in MD and RNFL thickness over time. To evaluate changes in visual field MD, we required at least five years of follow-up and ten visits. To evaluate changes in mean global RNFL thickness as measured on OCT, we required at least one year of follow-up and three visits. Predictors in multivariable mixed effects models of these progression markers included baseline age, follow-up in years, and presence of the e756del variant, and zygosity (i.e., homozygous or heterozygous). Longitudinal analyses were performed for all subjects in aggregate and also for glaucoma eyes only.

### Piezo1 gain-of-function (GOF) mouse models

Constitutive *Piezo1* gain-of-function mice (*Piezo1*^GOF^) were generated previously^11^. Mice were maintained on a C57BL/6 background. The experimental cohort was generated by mating two heterozygotes (*P1*^GOF/+^ X *P1*^GOF/+^), resulting in 11 wildtype *P1*^+/+^, 25 heterozygotes *P1*^GOF/+^, and 11 homozygotes *P1*^GOF/GOF^ ranging in age from 5 to 17 months. The mice were housed in a 12 hr light/dark cycle (lights on 6am to 6 pm) in a temperature-controlled room (24 degrees Celsius) with free access to food and water.

### Mouse IOP measurements

Mouse IOP measurements were obtained using an Icare Tonolab rebound tonometer for rodents (Icare Tonovet, Vantaa, Finland). All measurements were obtained between 10 a.m. and 4 p.m. Individual mice were briefly anesthetized immediately prior to and during IOP measurement. Anesthesia was performed with isoflurane (5% induction, 1–2% maintenance, Kent Scientific SomnoSuite, Torrington, CT, USA). IOP was measured according to the manufacturer’s protocol. Three consecutive measurements were measured and averaged for the reported value. A single examiner obtained all mouse IOP measurements and was masked to genotypes.

### Mouse RGC density

Mouse RGC density was measured by manual counting. Mice were deeply anesthetized with isoflurane prior to cervical dislocation. Eyes were immediately extracted and fixed in 4% paraformaldehyde (PFA) for 30 min. Retinas were then extracted from whole eyes and fixed for an additional 60 min in 4% PFA. RGCs were stained with anti-RBPMS antibody (Millipore: ABN1376) followed by a fluorescent secondary antibody for visualization. Retinas were whole-mounted and imaged at 40 × with a NikonC2 confocal microscope (Nikon Instruments, Melville, NY, USA). RGCs were counted from a max projection through the entire ganglion cell layer (GCL). RGCs were counted in three sample areas of 150 µm^2^ equidistant from the optic disk. An average of the three measurements was used for statistical comparisons. Genotypes were masked throughout this process.

### Statistical analyses

To evaluate associations between genotype and systemic phenotypes in humans, significance was determined based on two-sample *t*-tests for continuous variables and Fisher’s exact test for categorical variables. Genotypic associations with ocular phenotypes in humans were evaluated using linear mixed effects models with random intercepts to account for within-subject correlation. In addition to evaluating for any significant differences across all three groups (heterozygotes, homozygotes, and wildtypes), we also evaluated differences between groups in a pair-wise fashion. Multivariable mixed effects models were generated for changes in MD over time and changes in global mean RNFL thickness over time. Interaction terms between baseline age and years of follow-up and between the presence of e756del and years of follow-up were incorporated. All statistical analyses for human clinical data were conducted in R version 3.5.1^26^. For mouse phenotypes, a standard one-way analysis of variance (ANOVA) with a Tukey post-test for multiple comparisons was used to compare IOP and RGC density measured between wildtype, heterozygous and homozygous GOF mice. Statistical analyses for mouse data were conducted using Prism software (GraphPad Software Inc, San Diego, CA, USA). For all analyses in humans and in mice, statistical significance was defined as *p* < 0.05.

### Power calculations

Our analyses of human data were constrained by the number of individuals who had undergone whole genome sequencing and had sufficient ophthalmic data available. Thus, we evaluated our power given a set sample size using the *pwr* package in R^27^, which allows for multiple regression power calculation. Using the sample size of 683 individuals of African descent, a significance level of alpha = 0.05, f2 (effect size) of 0.02 (considered a small effect size by Cohen’s classic text^28^), we had 88.2% power to detect a statistically significant difference in phenotypes across 3 genotype groups (homozygous, heterozygous, wildtype). For evaluating visual field progression longitudinally, we only had 317 eyes from 170 patients with sufficient visual field data for those odels. With this sample size, we had 99.9% power to detect a modest effect size (f2 = 0.15) and 54.3% power to detect a small effect size (f2 = 0.02).

For power calculations in the mouse experiments, the R function *power.anova.test*^29^ was used to determine the power to detect a difference in mean RGC density of 15 cells per 150uM^2^ of mouse retina between groups of varying genotypes. This equated to a between-group variance of 225. With three groups (homozygous, heterozygous, and wildtype), a significance level designated at alpha = 0.05, and 11 mice per group, the study had a power of 91.4% to detect a statistically significant difference in RGC density.

## Results

### Allelic frequencies of Piezo1 gain-of-function variants among a multi-ethnic human cohort

In total, there were 1565 participants who underwent whole genome sequencing and identification of the *Piezo1* genotypic variants e756del and a1998v. Of these, based on racial admixture mapping of genome sequencing data, there were 683 (43.6%) individuals of African descent, 653 (41.7%) of European descent, 36 (2.3%) of Latino descent, 3 (0.2%) of East Asian descent, 2 (0.1%) of Central or South Asian descent, and 188 (12.0%) of other racial admixtures. Table 1 details the proportion of individuals with specific allele variants among the total study population, among individuals in the study population of African descent, and those of European descent. Allelic frequencies for each variant are also reported to reflect zygosity.

**Table 1:** Allelic frequencies of *Piezo1* gain-of-function variants in a multi-ethnic cohort of adults from the African Descent and Glaucoma Evaluation Study (ADAGES) and the Diagnostic Innovations in Glaucoma Study (DIGS).

	e756del variant		a1998v variant	
	Number (%)*	Allelic frequency^†^	Number (%)*	Allelic frequency^†^
Total population (N = 1565)^‡^	237 (15.14%)	8.66%	7 (0.45%)	0.22%
African descent (N = 683)	205 (30.01%)	17.42%	6 (0.88%)	0.44%
European descent (N = 653)	1 (0.15%)	0.08%	0 (0%)	0 (0%)

^*^Denotes number and percent of individuals with the variant (number with variant divided by number in population).

^†^Denotes frequency of the allele, accounting for some individuals being heterozygous and some individuals being homozygous.

^‡^Total population includes individuals of African descent, individuals of European descent, and a range of other racial admixtures.

Almost one-third of African descent individuals (205/683, 30.01%) had at least one *Piezo1* e756del allele. In contrast, there was only one individual heterozygous for e756del among 653 European descent individuals (allelic frequency of 0.08%). The a1998v variant was infrequent among African descent individuals (allelic frequency 0.44%), and there were none among European descent individuals. All individuals with a1998v also had the e756del variant.

### Associations between the e756del PIEZO1 variant and phenotypes in humans

Table 2 describes demographic and systemic phenotypes among e756del heterozygotes, homozygotes, and wild-type individuals of African descent. On average, e756del homozygotes (mean age 62.5 years) were slightly younger than heterozygotes (65.1 years) and wildtypes (65.7 years), although these differences were not statistically significant (p = 0.34). The gender distribution was also not significantly different among the different groups (p = 0.64). The e756del variant was not significantly associated with systolic blood pressure, diastolic blood pressure, or body mass index in this cohort (Table 2). No significant associations were found when looking at glaucoma subjects alone in a stratified analysis (Supplementary Table S1). Body mass index varied significantly based on genotype in healthy subjects (*p* < 0.01, Supplementary Table S2), but there were only two individuals without glaucoma who were homozygous for the e756del variant.

**Table 2:** General subject characteristics and systemic phenotypes based on *Piezo1* e756del variants among individuals of African descent. Data are presented as mean (95% confidence interval) for continuous variables and count (percentage) for categorical variables.

		e756 Deletion					
	A. Heterozygous	B. Homozygous	C. Wild-type	p-value	A vs. B	A vs. C	B vs. C
Age	*n* = 178	*n* = 27	*n* = 476				
	65.1 (63.5, 66.8)	62.5 (58.9, 66.1)	65.7 (64.7, 66.8)	0.343	0.180	0.555	0.086
**Gender**	*n* = 178	*n* = 27	*n* = 476				
Female	102 (57.3%)	13 (48.1%)	261 (54.8%)	0.642	0.410	0.597	0.554
Male	76 (42.7%)	14 (51.9%)	215 (45.2%)				
Mean systolic blood pressure	*n* = 161	*n* = 26	*n* = 431	0.909	0.683	0.995	0.663
	135.9 (132.8, 139.0)	134.3 (127.0, 141.6)	135.9 (134.2, 137.6)				
Mean diastolic blood pressure	*n* = 161	*n* = 26	*n* = 431	0.796	0.654	0.537	0.909
	82.6 (80.8, 84.4)	81.7 (78.4, 85.1)	81.9 (80.9, 82.9)				
**Body Mass Index**	*n* = 153	*n* = 25	*n* = 412	0.786	0.464	0.985	0.439
	29.9 (28.9, 30.8)	30.8 (28.5, 33.0)	29.9 (29.2, 30.5)				
**Patient classification**	*n* = 178	*n* = 27	*n* = 476				
Healthy	11 (6.2%)	2 (7.4%)	23 (4.8%)	0.844	0.626	0.912	0.385
Ocular Hypertension (OHT)	5 (2.8%)	1 (3.7%)	17 (3.6%)				
Glaucomatous Optic Nerve (GON)	45 (25.3%)	10 (37.0%)	112 (23.5%)				
Glaucomatous Visual Field Defect (GVFD)	12 (6.7%)	1 (3.7%)	36 (7.6%)				
GVFD & GON	105 (59.0%)	13 (48.1%)	288 (60.5%)				

There were several trends suggesting possible associations between the e756del mutation and ocular phenotypes in individuals of African descent, although these did not reach statistical significance (Table 3). Individuals homozygous for e756del had higher average IOP (25.4 mmHg) compared with heterozygotes (23.4 mmHg, p = 0.21) and compared with wildtypes (23.2 mmHg, p = 0.16). They also tended to have thicker corneas on average (mean CCT of 548.4 microns, compared with 532.3 for heterozygotes [p = 0.19] and 532.0 for wildtypes [p = 0.17]). The RNFL of e756del homozygotes tended to be thinner (mean 73.2 microns) than heterozygotes (80.3 microns, p = 0.15) and wildtypes (79.8 microns, p = 0.16), even though they were generally of younger age (Table 2). Additionally, optic nerve head capillary density measured on OCTA trended lower in both e756del heterozygotes (mean 40.6) and homozygotes (39.6) when compared with wildtypes (mean 43.3, p = 0.23 and p = 0.48, respectively), although these measurements were sparse; there were only two homozygous individuals with optic nerve head OCTA measurements available. In addition, there were no significant associations between e756del and ocular phenotypes in stratified analyses of glaucoma eyes only (Supplementary Table S3) and of healthy eyes only (Supplementary Table S4).

**Table 3:** Ocular phenotypes based on *Piezo1* e756del variants among eyes of individuals of African descent. Data are presented as mean (95% confidence interval) for continuous variables and count (percentage) for categorical variables. RNFL = retinal nerve fiber layer, GCC = ganglion cell complex, VF = visual field, ONH = optic nerve head.

	e756 Deletion			p-value	A vs. B	A vs. C	B vs. C
	A. Heterozygous	B. Homozygous	C. Wild-type				
IOP (Max)	*n* = 356	*n* = 54	*n* = 944	0.374	0.206	0.854	0.161
	23.4 (22.2, 24.5)	25.4 (22.5, 28.2)	23.2 (22.5, 23.9)				
AL	*n* = 193	*n* = 27	*n* = 466	0.959	0.789	0.856	0.842
	24.2 (23.8, 24.6)	24.1 (23.1, 25.0)	24.2 (23.9, 24.4)				
SE	*n* = 312	*n* = 45	*n* = 852	0.281	0.624	0.197	0.281
	− 0.45 (− 0.78, − 0.11)	− 0.21 (− 1.09, 0.66)	− 0.71 (− 0.91, − 0.50)				
CCT	*n* = 154	*n* = 20	*n* = 367	0.381	0.189	0.957	0.166
	532.3 (524.2, 540.4)	548.4 (525.8, 570.9)	532.0 (526.8, 537.2)				
RNFL Thickness (Spectralis)	*n* = 159	*n* = 32	*n* = 412	0.339	0.150	0.826	0.159
	80.3 (76.3, 84.3)	73.2 (64.4, 82.0)	79.8 (77.3, 82.2)				
GCC Thickness (Spectralis)	*n* = 29	*n* = 3	*n* = 73	0.413	0.278	0.664	0.196
	88.1 (81.1, 95.0)	76.3 (56.3, 96.2)	89.9 (85.5, 94.3)				
VF 24–2 MD	*n* = 288	*n* = 41	*n* = 772	0.611	0.963	0.337	0.717
	− 8.2 (− 9.5, − 6.9)	− 8.1 (− 11.6, − 4.7)	− 7.5 (− 8.3, − 6.7)				
Macula Superficial Density (Avanti)	*n* = 21	*n* = 2	*n* = 60	0.649	0.483	0.451	0.638
	40.5 (37.2, 43.9)	44.9 (33.3, 56.5)	42.0 (40.0, 44.1)				
ONH Capillary Density (Avanti)	*n* = 24	*n* = 3	*n* = 62	0.412	0.851	0.230	0.476
	40.6 (37.0, 44.3)	39.6 (30.0, 49.3)	43.3 (41.0, 45.6)				

To analyze glaucoma progression based on visual fields, we required at least five years of follow-up and ten visits, resulting in 170 subjects (113 without e756del, 57 with e756del) and 317 eyes (211 without e756del, 106 with e756del). Being heterozygous or homozygous for e756del carried negative coefficients in the model, suggesting that there was potentially a trend of greater decreases in mean deviation over time in eyes of individuals with the mutation, although this association did not reach statistical significance (Table 4). To analyze RNFL progression, we required at least one year of follow-up and three visits, resulting in 225 subjects (155 without e756del, 70 with e756del) and 421 eyes (287 without e756del, 134 with e756del). Again, e756del carried negative coefficients, suggesting a trend of the variant being associated with greater decreases in RNFL thickness over time (Table 4). This may have been influenced by different baseline RNFL values, given p < 0.01 for the intercept in the model. The interaction term between e756del homozygous status and follow-up had a negative coefficient (-0.45), suggesting a trend toward homozygotes having more rapid RNFL loss with follow-up (p = 0.20, see Table 4), even though they started with slightly thinner RNFLs. When longitudinal analyses were restricted to glaucoma eyes only, the e756del variant did not have any significant associations with changes in visual field mean deviation over time (Supplementary Table S5). However, there was again a negative coefficient for the interaction term between homozygosity for e756del with follow-up (-0.44, p = 0.25, Supplementary Table S6).

**Table 4:** Multivariable mixed effects models of the *Piezo1* e756del variant on longitudinal measures of glaucoma progression among individuals of African descent. Models were developed for changes in visual field mean deviation (MD) over time and changes in retinal nerve fiber layer (RNFL) thickness over time. Both homozygosity and heterozygosity were incorporated into the models, which were adjusted for baseline age and years of follow-up.

	Estimate (95% confidence interval)	*p* value
**Model of visual field MD (n = 170 patients, 317 eyes)**		
Intercept	− 1.36 (− 5.06, 2.35)	0.47
Baseline Age (years)	− 0.02 (− 0.09, 0.04)	0.48
Follow-up (years)	0.11 (− 0.06, 0.28)	0.21
e756del homozygous	− 1.32 (− 4.66, 2.03)	0.44
e756del heterozygous	− 0.34 (− 1.91, 1.22)	0.67
Baseline Age x Follow-up	0.00 (− 0.01, 0.00)	0.02
e756del homozygous x Follow-up	− 0.02 (− 0.18, 0.13)	0.77
e756del heterozygous x Follow-up	0.02 (− 0.05, 0.09)	0.55
**Model of RNFL thickness (n = 225 patients, 421 eyes)**		
Intercept	96.97 (84.35, 109.58)	< 0.01
Baseline Age (years)	− 0.20 (− 0.40, 0.00)	0.05
Follow-up (years)	− 1.35 (− 2.30, − 0.39)	< 0.01
e756del homozygous	− 6.38 (− 15.60, 2.83)	0.18
e756del heterozygous	− 1.40 (− 6.30, 3.49)	-0.56
Baseline Age x Follow-up	0.01 (− 0.01, 0.02)	0.26
e756del homozygous x Follow-up	− 0.45 (− 1.14, 0.23)	0.20
e756del heterozygous x Follow-up	0.07 (− 0.28, 0.43)	0.68

### Ocular phenotypes of Piezo1 gain-of-function mice

**Figure 1:**
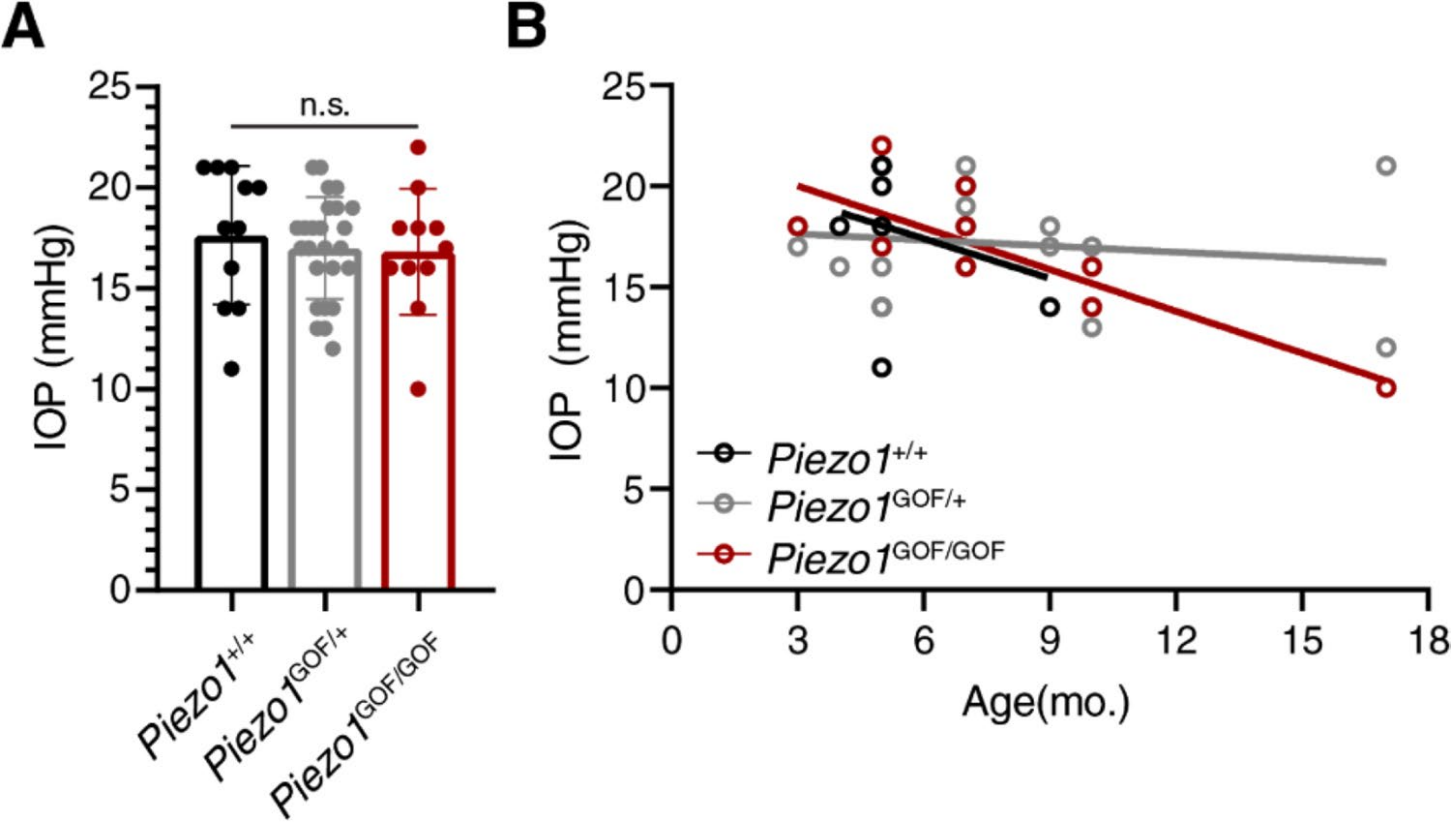
Intraocular pressure (IOP) in *Piezo1*^GOF^ mice. IOP was measured via tonometer in anesthetized mice (11 wildtype: Piezo1^+/+^, 25 heterozygotes: Piezo1^GOF/+/+^, and 11 homozygotes: Piezo1^GOF/GOF^). (**A**) No significant differences were observed between groups (one-way ANOVA with a Tukey post-test) (mean +/− s.d.). (**B**) The IOP measurements at various ages. A simple linear regression for each genotype is plotted for visualization. No obvious differences in the impact of age were noted.

To test the potential impact of a gain-of-function (GOF) *Piezo1* allele on IOP regulation and glaucoma we generated a cohort of constitutive Piezo1^GOF^ mice^11^ and assessed IOP and RGC density. We assayed 47 mice in total: 11 wildtype (Piezo1^+/+^), 25 heterozygotes (Piezo1^GOF/+^), and 11 homozygotes (Piezo1^GOF/GOF^). Measured IOP ranged from 10 to 22 mmHg (Fig. 1A). There were no significant differences in IOP between wildtype, heterozygous, and homozygous Piezo^GOF^ mice (wildtype mean = 17.6 ± 3.4 mmHg, heterozygous mean = 17.0 ± 2.5 mmHg, homozygous mean = 16.8 ± 3.1 mmHg). Given the age dependence of glaucoma-related phenotypes, we also assessed the relationship between age and IOP in our cohort (Fig. 1B). No significant differences between the genotypically distinct groups were observed, although insufficient numbers at each age were available to make a robust comparison.

**Figure 2:**
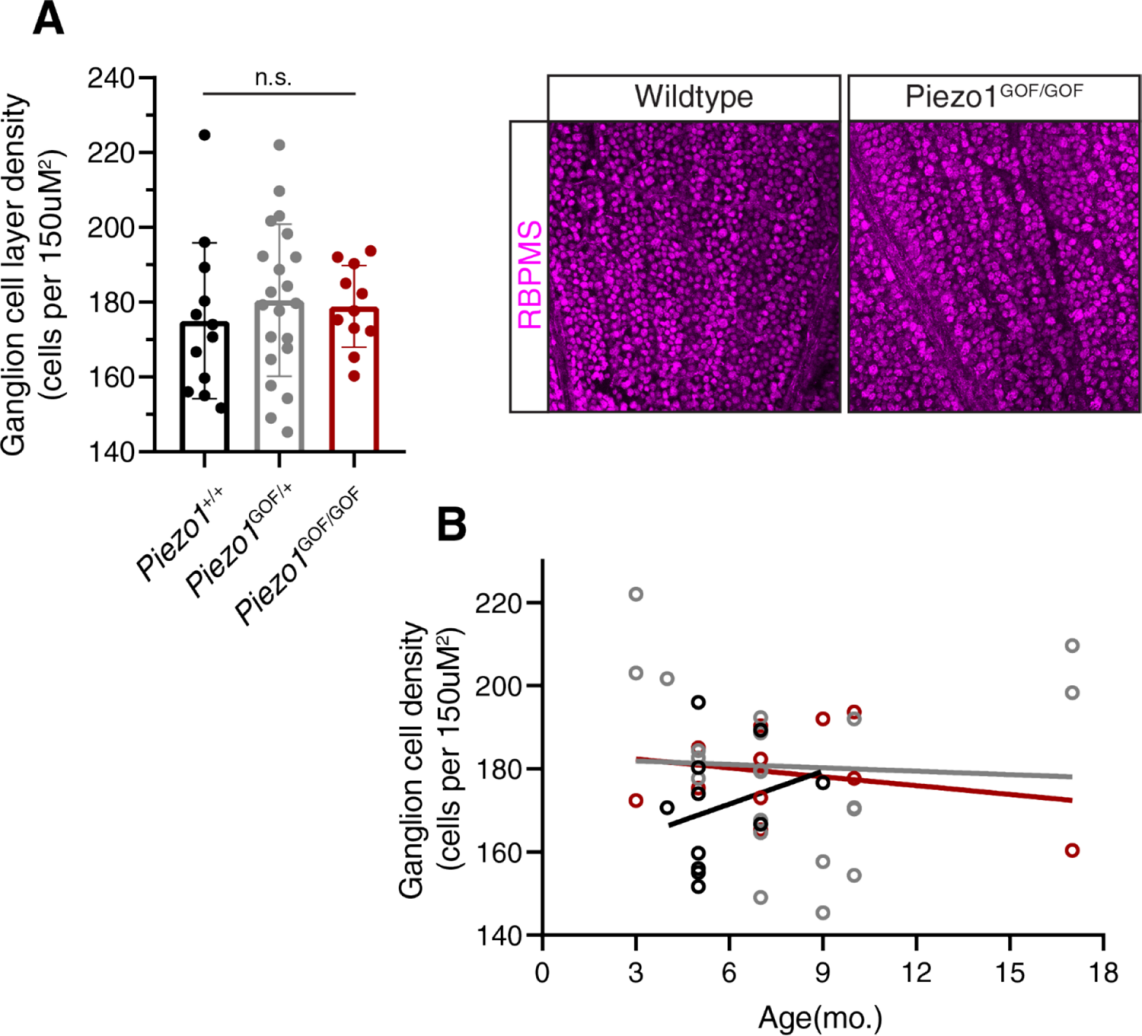
Ganglion cell density in *Piezo1*^GOF^ mice. Retinal ganglion cells were stained with an anti-RBPMS antibody (Millipore: ABN1376). Cell density was calculated in three 150 µm^2^ sample areas in each retina and the average was used per mouse. Example wildtype and homozygous staining is shown (RBPMS = magenta)(right). (**A**) No significant differences were observed between groups (one-way ANOVA with a Tukey post-test)(mean + /− s.d.). (**B**) No obvious differences in the impact of age were noted, but increased numbers would be required to make any robust comparisons. A simple linear regression per genotype is shown for visualization.

To determine the impact of increased *Piezo1* function on RGC survival, we measured RGC density in our Piezo1^GOF^ cohort (Fig. 2A). Similar to the IOP measurements, we saw no significant differences in RGC density between groups (wildtype mean = 175 ± 20.8 cells per 150 uM^2^, het mean = 180 ± 20.3, homo mean = 179 ± 10.9). Additionally, no obvious differences in the impact of age on RGC density were observed (Fig. 2B). As mentioned previously, numbers within each age group were insufficient for robust statistical comparisons.

## Discussion

In this study, we report our analyses of *Piezo1* genotypic variants with glaucoma-related phenotypes in humans and phenotypic associations in mice. Although we did not detect clear associations between *Piezo1* gain-of-function mutations and glaucoma-related phenotypes, several important insights were generated.

First, the allelic frequencies of both the e756del and the a1998v variants in this study population were consistent with previously reported frequencies. The allelic frequency for e756del among African descent individuals here was 17.42%, while Ma et al. reported the allelic frequency as 18% in a separate cohort^11^. Similarly, the allelic frequency of a1998v among African descent individuals here was 0.44%, whereas prior reports for this were estimated at 0.8%^11^. Almost one-third of the African descent individuals in this cohort had at least 1 allele with the e756del variant. While we did not find evidence for this mutation to have a role in glaucoma phenotypes, this common variant may have implications for other disease processes, particularly those in which African descent individuals are disproportionately affected. Although our analyses did not demonstrate significant associations with systemic conditions such as blood pressure, further studies of systemic phenotypes are warranted to better understand the health consequences of this mutation, particularly because prior studies in mice demonstrated a clear role of PIEZOs in baroreception^10^ and would predict an impact on blood pressure-related phenotypes in humans.

With the e756del variant being this common, we did not expect strong associations for clinical phenotypes in humans. Primary open-angle glaucoma is known to be a complex disease with multiple environmental, social, genetic, and epigenetic factors, and how these factors interact to modulate disease risk has yet to be fully elucidated^30–35^. As a result, we knew that single genetic variants in *Piezo1* were unlikely to show statistically significant associations with glaucoma phenotypes in humans, even though we were sufficiently powered to detect even small effect sizes in most phenotypes (the exception being visual fields, where we were constrained by the amount of available data of sufficient quality for longitudinal modeling). Our goal was to explore whether interesting trends arose that would warrant further investigation with mouse experimental studies, where there would be less environmental and genetic variability. And indeed, our analyses of human data revealed that e756del homozygotes tended to have higher IOPs in healthy subjects and those with glaucoma (although this may have been driven in part by thicker corneas) and thinner RNFLs (despite younger age). Mixed effects models of changes in RNFL thickness over time in both the cohort as a whole and among glaucoma subjects only suggested trends of e756del homozygosity being associated with more rapid decreases in RNFL thickness over time compared with wild-type (Table 4, Supplementary Table S6). While the p-values did not reach the commonly used threshold of p < 0.05, even in the p ~ 0.20 range, we were motivated to pursue additional investigation using *Piezo1* mouse models.

There were some limitations associated with the clinical data from humans. First, there is a known effect of aging on changes in visual field and RNFL over time, among both glaucomatous and healthy eyes^36–39^. We attempted to account for this by including baseline age, follow-up, and interaction terms between the genetic variants and length of follow-up in the mixed effects models. However, it is still possible there was some confounding from aging-related effects. Second, we were interested to see whether there might be differential effects for glaucomatous eyes and for healthy eyes and therefore conducted stratified analyses within each of these sub-groups. However, these sub-groups consisted of smaller samples and were likely under-powered to detect differences across genotypes. Finally, the number of eyes with OCTA testing results available among individuals with the e756del variant was low (< 25), so these analyses were also relatively underpowered.

Our experiments in mice showed no significant impact of increased *Piezo1* activity on IOP or ganglion cell numbers. However, there were several limitations to these experiments that are worth noting. Due to the small size of the eyes, in part, IOP measurements in mice are less accurate and reproducible than in humans. Additionally, our mice were anesthetized during IOP measurement, which is known to impact IOP^40^. Further, our measurement of ganglion cell numbers was done by sampling areas around the retina without regard for nasal/temporal/dorsal/lateral location of the sample. This method would miss any regional impact of the *Piezo1*^*GOF*^ allele in the ganglion cell layer. With a more complete analysis of retinal ganglion cell density (throughout the entire retina), there may be an impact of the *Piezo1*^*GOF*^ allele. Third, while our mouse cohort was large (47 mice aged 5 to 17 months) and sufficiently powered to detect statistically significant differences across the entire cohort, there were not sufficient numbers within different age groups to make meaningful comparisons of potential effects by age. This is especially true for the older mice, where we lacked any wildtype mice for comparison. Since glaucoma is known to be acutely dependent upon age in humans, it will be important to more thoroughly investigate aged mice with sufficient numbers. Lastly, mouse eyes and retinas differ considerably from humans. While the mouse serves as a useful tool for glaucoma research, it is possible that systems critical in humans do not exist in mice. With all of that said, our work suggests that increased *Piezo1* activity in mice does not lead to glaucoma-like phenotypes.

Taken together, our work confirms that the *Piezo1* e756del variant is a frequent polymorphism present in individuals of African descent, but that this specific variant may be unlikely to have a significant role in influencing glaucoma phenotypes, based on our analyses of relatively large cohorts of both humans and mice that were sufficiently powered to detect these phenotypic differences. However, given that PIEZO1 channels have been found in the trabecular meshwork of human eyes^17^, and that glaucoma is clearly a pressure-sensitive disease, further investigations of *Piezo1* variants in glaucoma are still warranted. This is especially true because the *Piezo1* locus is not easily studied in genome-wide association studies (GWAS), which mainly use single nucleotide polymorphisms to determine associations and encounter challenges when evaluating more complex loci. For example, the locus containing e756del has multiple short tandem repeats, and therefore imputation of this mutation into current GWAS datasets is not straightforward^11^. Analyzing *Piezo1* variants in tandem with other known loci identified from prior GWAS analyses in glaucoma may yield further insights. Another potential future direction would be to further evaluate the effects of *Piezo1* on capillary density measured with OCTA, an imaging modality that can provide additional information about early detection of glaucomatous changes^41–43^. At the time of this analysis, few individuals in our clinical cohort had undergone OCTA testing, so we were not able to generate sufficient power for comparison. However, this may be a promising route of investigation in the future with additional testing. In summary, much work remains to continue developing our understanding of PIEZO1 and mechanotransduction in the pathophysiology and management of glaucoma and other conditions.

## Supplementary information

Supplementary Information

## References

[1] Tham Y-C (2014). Global prevalence of glaucoma and projections of glaucoma burden through 2040: a systematic review and meta-analysis. Ophthalmology.

[2] Glaucoma, Open-angle | National Eye Institute. https://www.nei.nih.gov/eyedata/glaucoma.

[3] Weinreb RN, Aung T, Medeiros FA (2014). The pathophysiology and treatment of glaucoma: a review. JAMA.

[4] Gracitelli CPB (2015). Association between progressive retinal nerve fiber layer loss and longitudinal change in quality of life in glaucoma. JAMA Ophthalmol..

[5] Coste B (2010). Piezo1 and Piezo2 are essential components of distinct mechanically activated cation channels. Science.

[6] Woo S-H (2014). Piezo2 is required for Merkel-cell mechanotransduction. Nature.

[7] Ranade SS (2014). Piezo2 is the major transducer of mechanical forces for touch sensation in mice. Nature.

[8] Chesler, A. T. *et al.* The Role of PIEZO2 in Human Mechanosensation. (2016) 10.1056/NEJMoa1602812.

[9] Woo S-H (2015). Piezo2 is the principal mechanotransduction channel for proprioception. Nat. Neurosci..

[10] Zeng W-Z (2018). PIEZOs mediate neuronal sensing of blood pressure and the baroreceptor reflex. Science.

[11] Ma S (2018). Common PIEZO1 allele in African populations causes RBC dehydration and attenuates plasmodium infection. Cell.

[12] Cahalan SM (2015). Piezo1 links mechanical forces to red blood cell volume. eLife.

[13] Ranade SS (2014). Piezo1, a mechanically activated ion channel, is required for vascular development in mice. Proc. Natl. Acad. Sci..

[14] Choi HJ, Sun D, Jakobs TC (2015). Astrocytes in the optic nerve head express putative mechanosensitive channels. Mol. Vis..

[15] Koser DE (2016). Mechanosensing is critical for axon growth in the developing brain. Nat. Neurosci..

[16] Song Y (2019). The mechanosensitive ion channel piezo inhibits axon regeneration. Neuron.

[17] Tran VT, Ho PT, Cabrera L, Torres JE, Bhattacharya SK (2014). Mechanotransduction channels of the trabecular meshwork. Curr. Eye Res..

[18] Tielsch JM (1991). Racial variations in the prevalence of primary open-angle glaucoma. The Baltimore Eye Survey. JAMA.

[19] Leske MC (2008). Risk factors for incident open-angle glaucoma: the Barbados Eye Studies. Ophthalmology.

[20] Leske MC (2007). Nine-year incidence of open-angle glaucoma in the Barbados eye studies. Ophthalmology.

[21] Sample PA (2009). The African Descent and Glaucoma Evaluation Study (ADAGES): design and baseline data. Arch. Ophthalmol. Chic. Ill.

[22] Girkin CA (2010). African Descent and Glaucoma Evaluation Study (ADAGES): II. Ancestry differences in optic disc, retinal nerve fiber layer, and macular structure in healthy subjects. Arch. Ophthalmol. Chic. Ill.

[23] Racette L (2010). African Descent and Glaucoma Evaluation Study (ADAGES): III. Ancestry differences in visual function in healthy eyes. Arch. Ophthalmol. Chic. Ill.

[24] Zangwill LM (2019). The African Descent and Glaucoma Evaluation Study (ADAGES) III: contribution of genotype to glaucoma phenotype in African Americans: study design and baseline data. Ophthalmology.

[25] Telenti A (2016). Deep sequencing of 10,000 human genomes. Proc. Natl. Acad. Sci. U. S. A..

[26] R Core Team. R: A language and environment for statistical computing. (2013).

[27] Champely, S. *et al. pwr: Basic Functions for Power Analysis*. (2020).

[28] Cohen J (1988). Statistical Power Analysis for the Behavioral Sciences.

[29] power.anova.test function | R Documentation. https://www.rdocumentation.org/packages/stats/versions/3.6.2/topics/power.anova.test.

[30] Gauthier AC, Liu J (2017). Epigenetics and Signaling Pathways in Glaucoma. BioMed Res. Int..

[31] Wiggs JL, Pasquale LR (2017). Genetics of glaucoma. Hum. Mol. Genet..

[32] Genetics of Glaucoma in People of African Descent (GGLAD) Consortium *et al.* Association of Genetic Variants With Primary Open-Angle Glaucoma Among Individuals With African Ancestry. *JAMA* **322**, 1682–1691 (2019). 10.1001/jama.2019.16161 PMC6865235 31688885

[33] Shiga Y (2018). Genome-wide association study identifies seven novel susceptibility loci for primary open-angle glaucoma. Hum. Mol. Genet..

[34] Khawaja AP (2018). Genome-wide analyses identify 68 new loci associated with intraocular pressure and improve risk prediction for primary open-angle glaucoma. Nat. Genet..

[35] Doucette LP, Rasnitsyn A, Seifi M, Walter MA (2015). The interactions of genes, age, and environment in glaucoma pathogenesis. Surv. Ophthalmol..

[36] Tatham AJ, Medeiros FA (2017). Detecting structural progression in glaucoma with optical coherence tomography. Ophthalmology.

[37] Belghith A (2016). Structural change can be detected in advanced-glaucoma eyes. Invest. Ophthalmol. Vis. Sci..

[38] Wu Z (2017). Impact of normal aging and progression definitions on the specificity of detecting retinal nerve fiber layer thinning. Am. J. Ophthalmol..

[39] Bowd C (2002). Imaging of the optic disc and retinal nerve fiber layer: the effects of age, optic disc area, refractive error, and gender. J. Opt Soc. Am. A. Opt. Image Sci. Vis..

[40] Wang W-H, Millar JC, Pang I-H, Wax MB, Clark AF (2005). Noninvasive Measurement of Rodent Intraocular Pressure with a Rebound Tonometer. Invest. Ophthalmol. Vis. Sci..

[41] Hou H (2019). Macula vessel density and thickness in early primary open-angle glaucoma. Am. J. Ophthalmol..

[42] Lu P (2020). Quantitative analysis of microvasculature in macular and peripapillary regions in early primary open-angle glaucoma. Curr. Eye Res..

[43] Bowd C (2020). gradient boosting classifiers combining vessel density and tissue thickness measurements for classifying early to moderate glaucoma. Am. J. Ophthalmol..

