# Supplementary material for: Investigation of associations between Piezo1 mechanoreceptor gain-of-function variants and glaucoma-related phenotypes in humans and mice
Source: Sci Rep. 2020 Nov 4;10:19013. doi: 10.1038/s41598-020-76026-0 (PMC7643131; doi:10.1038/s41598-020-76026-0)
Supplement: Supplementary file 1 — Supplementary Information [file 41598_2020_76026_MOESM1_ESM.pdf]

Supplemental Material

Investigation of associations between *Piezo1* mechanoreceptor gain-of-function variants and glaucoma-related phenotypes in humans and mice

Sally L. Baxter, MD, MSc[1,2a]; William T. Keenan, PhD[3a]; Argus J. Athanas, PhD[2]; James A. Proudfoot, MS[1]; Linda M. Zangwill, PhD[1]; Radha Ayyagari, PhD[1]; Jeffrey M. Liebmann, MD[4]; Christopher A. Girkin, MD[5]; Ardem Patapoutian, PhD[3]; Robert N. Weinreb, MD*[1]

[a]Contributed equally

[1]Hamilton Glaucoma Center, Viterbi Family Department of Ophthalmology, and Shiley Eye Institute, University of California San Diego (UCSD), La Jolla, CA
[2]Health Department of Biomedical Informatics, UCSD, La Jolla, CA
[3]Scripps Research Institute, La Jolla, CA
[4]Bernard and Shirlee Brown Glaucoma Research Laboratory, Edward S. Harkness Eye Institute, Columbia University Irving Medical Center, New York, NY
[5]Department of Ophthalmology and Vision Sciences, Callahan Eye Hospital, University of Alabama at Birmingham, Birmingham, AL

***Corresponding Author:**
Robert N. Weinreb, MD
9415 Campus Point Drive, MC0946
La Jolla, CA 92093
rweinreb@health.ucsd.edu
T: (858) 534-8824 | F: (858) 534-1625

Table S1. General subject characteristics and systemic phenotypes based on *Piezo1* e756del variants among individuals of African descent – stratified analysis for glaucoma subjects only. Data are presented as mean (95% confidence interval) for continuous variables and count (percentage) for categorical variables.

	e756 Deletion						
	A. Heterozygous	B. Homozygous	C. Wild-type	p-value	A vs. B	A vs. C	B vs. C
**Age**	n = 105	n = 13	n = 288				
	67.0 (65.0, 69.0)	64.3 (58.4, 70.2)	68.0 (66.7, 69.3)	0.373	0.373	0.386	0.207
**Gender**	n = 105	n = 13	n = 288				
Female	56 (53.3%)	8 (61.5%)	149 (51.7%)	0.770	0.769	0.820	0.577
Male	49 (46.7%)	5 (38.5%)	139 (48.3%)				
**Mean Systolic Blood Pressure**	n = 95	n = 13	n = 262				
	137.9 (133.3, 142.6)	130.4 (117.5, 143.2)	135.8 (133.6, 138.0)	0.370	0.250	0.414	0.381
**Mean Diastolic Blood Pressure**	n = 95	n = 13	n = 262				
	83.2 (80.5, 85.9)	80.5 (74.8, 86.2)	81.9 (80.7, 83.0)	0.483	0.365	0.358	0.619
**Body Mass Index**	n = 93	n = 13	n = 258				
	29.6 (28.3, 30.8)	30.4 (27.4, 33.4)	29.7 (28.9, 30.5)	0.914	0.597	0.890	0.628
**Patient Classification**	n = 105	n = 13	n = 288				
Healthy	0 (0.0%)	0 (0.0%)	0 (0.0%)	-	1.000	1.000	1.000
Ocular Hypertension (OHT)	0 (0.0%)	0 (0.0%)	0 (0.0%)				
Glaucomatous Optic Nerve (GON)	0 (0.0%)	0 (0.0%)	0 (0.0%)				
Glaucomatous Visual Field Defect	0 (0.0%)	0 (0.0%)	0 (0.0%)				
GVFD & GON	105 (100.0%)	13 (100.0%)	288 (100.0%)				

Table S2. General subject characteristics and systemic phenotypes based on *Piezo1* e756del variants among individuals of African descent – stratified analysis for healthy subjects only. Data are presented as mean (95% confidence interval) for continuous variables and count (percentage) for categorical variables.

	e756 Deletion						
	A. Heterozygous	B. Homozygous	C. Wild-type	p-value	A vs. B	A vs. C	B vs. C
**Age**	n = 11	n = 2	n = 23				
	59.4 (51.6, 67.2)	55.8 (-11.6, 123.3)	54.9 (49.5, 60.3)	0.601	0.631	0.314	0.893
**Gender**	n = 11	n = 2	n = 23				
Female	7 (63.6%)	0 (0.0%)	16 (69.6%)	0.145	0.192	1.000	0.120
Male	4 (36.4%)	2 (100.0%)	7 (30.4%)				
**Mean Systolic Blood Pressure**	n = 11	n = 2	n = 23				
	131.2 (119.9, 142.4)	138.8 (98.7, 178.9)	127.2 (123.5, 130.8)	0.308	0.234	0.467	0.103
**Mean Diastolic Blood Pressure**	n = 11	n = 2	n = 23				
	81.0 (75.4, 86.7)	85.6 (60.9, 110.4)	75.9 (73.3, 78.4)	0.041*	0.206	0.089	0.053
**Body Mass Index**	n = 9	n = 2	n = 15				
	28.4 (24.8, 31.9)	40.6 (31.9, 49.4)	28.1 (25.4, 30.7)	0.005**	<0.001**	0.877	<0.001**
**Patient Classification**	n = 11	n = 2	n = 23				
Healthy	11 (100.0%)	2 (100.0%)	23 (100.0%)	-	1.000	1.000	1.000
Ocular Hypertension (OHT)	0 (0.0%)	0 (0.0%)	0 (0.0%)				
Glaucomatous Optic Nerve (GON)	0 (0.0%)	0 (0.0%)	0 (0.0%)				
Glaucomatous Visual Field Defect	0 (0.0%)	0 (0.0%)	0 (0.0%)				
GVFD & GON	0 (0.0%)	0 (0.0%)	0 (0.0%)				

Table S3. Ocular phenotypes based on *Piezo1* e756del variants among individuals of African descent – stratified analysis for glaucoma eyes only. Data are presented as mean (95% confidence interval) for continuous variables and count (percentage) for categorical variables. IOP=intraocular pressure, AL=axial length, SE=spherical equivalent, CCT=central corneal thickness, RNFL = retinal nerve fiber layer, GCC = ganglion cell complex, VF = visual field, ONH = optic nerve head.

	e756 Deletion		
	No	Yes	p-value
**IOP (Max)**	$n = 572$<br>23.7 (22.9, 24.6)	$n = 236$<br>24.7 (23.3, 26.0)	0.256
**AL**	$n = 247$<br>24.3 (23.9, 24.7)	$n = 116$<br>24.4 (23.9, 25.0)	0.717
**SE**	$n = 511$<br>−0.69 (−0.95, −0.44)	$n = 212$<br>−0.46 (−0.86, −0.07)	0.344
**CCT**	$n = 177$<br>531.9 (524.4, 539.4)	$n = 94$<br>538.2 (527.8, 548.6)	0.340
**RNFL Thickness (Spectralis)**	$n = 211$<br>70.3 (67.4, 73.2)	$n = 95$<br>68.7 (64.4, 73.0)	0.562
**GCC Thickness (Spectralis)**	$n = 36$<br>83.4 (76.9, 89.8)	$n = 19$<br>79.6 (70.7, 88.5)	0.502
**VF 24-2 MD**	$n = 521$<br>−9.5 (−10.4, −8.5)	$n = 213$<br>−10.9 (−12.4, −9.4)	0.124
**Macula Superficial Density (Avanti)**	$n = 32$<br>39.6 (36.9, 42.3)	$n = 14$<br>38.0 (34.0, 41.9)	0.504
**ONH Capillary Density (Avanti)**	$n = 32$<br>39.3 (36.5, 42.1)	$n = 18$<br>37.9 (34.1, 41.6)	0.548

Table S4. Ocular phenotypes based on *Piezo1* e756del variants among individuals of African descent – stratified analysis for healthy eyes only. Data are presented as mean (95% confidence interval) for continuous variables and count (percentage) for categorical variables. IOP=intraocular pressure, AL=axial length, SE=spherical equivalent, CCT=central corneal thickness, RNFL = retinal nerve fiber layer, GCC = ganglion cell complex, VF = visual field, ONH = optic nerve head.

	e756 Deletion		
	No	Yes	p-value
**IOP (Max)**	$n = 46$<br>16.5 (15.7, 17.3)	$n = 26$<br>16.6 (15.6, 17.6)	0.862
**AL**	$n = 46$<br>23.6 (23.2, 24.0)	$n = 26$<br>23.4 (22.8, 24.0)	0.618
**SE**	$n = 46$<br>−0.42 (−1.03, 0.20)	$n = 26$<br>0.07 (−0.75, 0.88)	0.358
**CCT**	$n = 46$<br>532.0 (519.8, 544.3)	$n = 26$<br>534.7 (518.4, 551.0)	0.800
**RNFL Thickness (Spectralis)**	$n = 46$<br>97.1 (92.7, 101.4)	$n = 26$<br>98.9 (93.1, 104.7)	0.618
**GCC Thickness (Spectralis)**	$n = 10$<br>100.0 (97.8, 102.3)	$n = 2$<br>98.9 (93.8, 103.9)	0.700
**VF 24-2 MD**	$n = 46$<br>−0.5 (−1.0, 0.1)	$n = 26$<br>−0.6 (−1.3, 0.2)	0.806
**Macula Superficial Density (Avanti)**	$n = 9$<br>44.0 (39.1, 48.8)	$n = 2$<br>44.9 (34.4, 55.4)	0.889
**ONH Capillary Density (Avanti)**	$n = 10$<br>49.0 (46.2, 51.7)	$n = 2$<br>47.6 (41.5, 53.7)	0.715

Table S5. Multivariable mixed effects model of the *Piezo1* e756del variant on visual field mean deviation over time among individuals of African descent – stratified analysis of glaucoma eyes only. We required at least five years of follow-up and ten visits in the analysis that follows. This led to a sample size of n = 90 subjects (58 without e756 deletion and 32 with e756 deletion) and 165 eyes (105 without e756 deletion and 60 with e756 deletion).

	Estimate	Std. Error	95% CI	t-value	p-value
(Intercept)	−5.00	3.18	(−11.24, 1.25)	−1.57	0.121
Baseline Age	0.02	0.05	(−0.09, 0.12)	0.33	0.744
Follow-Up (Years)	−0.14	0.14	(−0.41, 0.14)	−0.98	0.330
e756 Deletion: Homozygous	−1.75	2.69	(−7.01, 3.52)	−0.65	0.518
e756 Deletion: Heterozygous	−0.77	1.34	(−3.39, 1.86)	−0.57	0.568
Baseline Age × Follow-Up (Years)	−0.00	0.00	(−0.01, 0.00)	−0.26	0.796
e756 Deletion: Homozygous × Follow-Up (Years)	−0.01	0.12	(−0.24, 0.22)	−0.07	0.941
e756 Deletion: Heterozygous × Follow-Up (Years)	0.05	0.06	(−0.06, 0.16)	0.86	0.393

Table S6. Multivariable mixed effects model of the *Piezo1* e756del variant on global mean retinal nerve fiber layer (RNFL) thickness over time among individuals of African descent – stratified analysis of glaucoma eyes only. We required at least one year of follow-up and three visits in the analysis that follows. This led to a sample size of n = 124 subjects (83 without e756 deletion and 41 with e756 deletion) and 230 eyes (151 without e756 deletion and 79 with e756 deletion).

	Estimate	Std. Error	95% CI	t-value	p-value
(Intercept)	72.36	7.80	(57.07, 87.65)	9.27	<0.001***
Baseline Age	0.07	0.12	(−0.17, 0.31)	0.57	0.567
Follow-Up (Years)	−2.05	0.56	(−3.15, −0.95)	−3.66	<0.001***
e756 Deletion: Homozygous	−3.34	5.46	(−14.03, 7.36)	−0.61	0.542
e756 Deletion: Heterozygous	−1.74	3.02	(−7.67, 4.19)	−0.58	0.566
Baseline Age × Follow-Up (Years)	0.02	0.01	(0.00, 0.04)	2.27	0.024*
e756 Deletion: Homozygous × Follow-Up (Years)	−0.44	0.39	(−1.20, 0.31)	−1.15	0.253
e756 Deletion: Heterozygous × Follow-Up (Years)	0.12	0.21	(−0.29, 0.53)	0.59	0.557
